# Widespread DNA double strand break and repair events excise most of APOE exon 4 in Alzheimer's disease

**DOI:** 10.1002/alz70855_105919

**Published:** 2025-12-24

**Authors:** Julianna N Brutman, Eli J Kaufman, Evangelos Nizamis, Tina Busald, Samuel Smukowski, Marissa J de Leon, Corbin S.C. Johnson, Caitlin S Latimer, Daniel D Child, Thomas D. Bird, Katherine E Prater, Suman Jayadev, Paul N Valdmanis

**Affiliations:** ^1^ University of Washington, Seattle, WA, USA; ^2^ GRECC VA Puget Sound, Seattle, WA, USA

## Abstract

**Background:**

*APOE* genotype status is the strongest common risk factor for Alzheimer's disease (AD), yet the biological mechanisms that underlie this pathogenesis are still unclear. Under normal circumstances, neuronal double‐stranded breaks (DSBs) facilitate memory formation. However, DSBs also occur in early AD pathology, though a consistent pattern or pathological mechanism has not been observed.

**Methods:**

We performed analysis of single‐nuclei RNA sequencing (snRNAseq) data from published unsorted and PU.1 sorted microglia from human post‐mortem dorsolateral prefrontal cortex. We further analyzed ∼36,000 DNA samples from the Alzheimer's Disease Sequencing Project to confirm presence of reads with gaps in DNA sequence.

**Results:**

We found 1,999 sequence reads from ∼36,000 samples from the Alzheimer's Disease Sequencing Project exhibit DNA double‐strand break and repair events specifically at *APOE*. These DSBs removed an average of 334bp of sequence and were primarily located in the last exon of *APOE*. Several DSBs originated or terminated at *APOE‐ε4, APOE‐ε2*, or *APOE*‐Christchurch variants, or removed these variants entirely. Curiously, DSBs present in the coding region of *APOE* preserved the protein reading frame 96% of the time (*n* = 1,816 reads spread across 56 different breakpoints) compared to 22% of reads that extended past the 3′UTR (*p* <2.2×10^‐16^ by chi‐square test). Amazingly, these DSB events are also transcribed: we detected 1,223 sequence reads from 22 individuals from snRNAseq data with the same breakpoints and loss of the *APOE* sequence, averaging 337bp. Split‐reads lacked canonical splice donor and acceptor sites suggesting they originated at the DNA level. Split‐reads were present most frequently in astrocytes, followed by microglia with only rare instances detected in neurons. Importantly, 7 of 12 AD samples and 0 of 10 controls had split‐reads that originated from a 13bp segment immediately after the *APOE‐ε4* SNP rs429358 (*p* = 0.0053 by Fisher's exact test).

**Conclusion:**

Our data reveal that *APOE* is subject to widespread double‐strand break and repair events excising key *APOE* risk variants in the process. These data have profound implications for *APOE* function and our understanding of the mechanism underlying *APOE* risk.

